# Oral administration of East Asian herbal medicine for rheumatoid arthritis: A protocol for systematic review and meta-analysis

**DOI:** 10.1097/MD.0000000000028819

**Published:** 2022-02-11

**Authors:** Hee-Geun Jo, Ho-Sueb Song, Donghun Lee

**Affiliations:** a Department of Bioinformatics and Statistics, Graduate School of Korea National Open University, 86 Daehak-ro, Jongro-gu, Seoul, Republic of Korea; b Department of Acupuncture & Moxibustion Medicine, College of Korean Medicine, Gachon University 1342 Seongnamdae-ro, Sujeong-gu, Seongnam, Republic of Korea; c Department of Herbal Pharmacology, College of Korean Medicine, Gachon University, 1342 Seongnamdae-ro, Sujeong-gu, Seongnam, Republic of Korea.

**Keywords:** arthralgia, complementary and alternative medicine, East Asian herbal medicine, meta-analysis, rheumatoid arthritis, systematic review

## Abstract

**Background:**

Rheumatoid arthritis (RA) is a chronic, inflammatory, and painful joint disease. The aim of this review is to systematically evaluate the efficacy and safety of oral administration East Asian herbal medicine monotherapy for inflammatory pain of RA, and to explore core herb material information based on collected data.

**Methods:**

A comprehensive literature search will be conducted in 11 electronic databases including PubMed, Cochrane Library, Cumulative Index to Nursing & Allied Health Literature, Excerpta Medica database, Korean Studies Information Service System, Research Information Service System Oriental Medicine Advanced Searching Integrated System, Korea Citation Index, Chinese National Knowledge Infrastructure Database, Wanfang data, citation information by NII for randomized controlled trials from their inception until October 13, 2021. Statistical analysis will be performed in the software R version 4.1.1. and R studio program using the default settings of the “meta” and “metafor” package. When heterogeneity in studies is detected, the cause will be identified through subgroup analysis. Methodological quality will be assessed independently using the revised tool for risk of bias in randomized trials (Rob 2.0).

**Results:**

This study will provide more comprehensive and specific evidence of East Asian herbal medicine monotherapy for RA pain management.

**Conclusions:**

Based on the results of this review, it is expected that the efficacy and safety of East Asian herbal medicine for inflammatory pain of RA may be confirmed. In addition, it will be possible to derivation of a core herb material information related to this research topic through additional data mining.

**Ethics and dissemination:**

There are no ethical issues as there are no primary data collected by directly recruiting subjects. The results of this review will be reported in a peer-reviewed scientific journal.

**PROSPERO registration number:**

CRD42021273643

## 1 Introduction

Rheumatoid arthritis (RA) is a chronic, inflammatory, and painful joint disease. The worldwide prevalence of RA between 1980 and 2019 was 460 per 100,000 population.^[1]^ RA causes disability and inability to work due to symptoms such as progressive inflammatory conditions and pain.^[2]^ If RA is not treated early and adequately, extra-articular features such as rheumatoid nodules or rheumatoid vasculitis may be induced.^[3,4]^ Furthermore, as a result of chronic inflammation, several comorbidities such as various cardiovascular diseases are commonly detected in patients with RA for a long time, which leads to a direct cause of mortality.^[5]^

Even though RA is incurable, different forms of conventional medicine (CM) have been created and are helping to regulate the condition. Anti-inflammation medications such as traditional analgesics like non-steroidal anti-inflammatory drugs (NSAIDs) and glucocorticoids like have been used for a long time, but disease-modifying antirheumatic drugs have lately been suggested as first-line treatment.^[6,7]^ However, given the disease's long-term course, the discovery of safer medications is still important. Even NSAIDs, which are thought to be the safest, reinforce this notion by reporting considerable numbers of adverse events such as gastrointestinal problems, renal impairment, and cardiovascular events.^[8,9]^ Meanwhile, disease-modifying antirheumatic drugs are the most advanced RA treatment at the moment, but cannot guarantee a perfectly consistent impact due to the strong variability of RA patients. Hence, a challenge related to drug discovery for RA will be the search for safer materials that can reduce adverse events during long-term dosage and alternative materials for patients who do not respond to conventional medication. East Asian herbal medicine (EAHM) could be a useful candidate for RA treatment that meets aforementioned objective. In East Asian nations such as China, Korea, and Japan, 500 to 1000 EAHM materials are commonly listed in pharmacopeia and are still actively utilized as medications for the treatment of rheumatic and musculoskeletal illnesses such.^[10–13]^

Based on previous study knowledge and challenge awareness, the authors set the following research objectives:

1. A systematic literature review of the maximum searchable range of clinical trials that compared the effects of EAHM monotherapy and conventional drugs was performed to evaluate whether EAHM is a material worth continuously using and studying for inflammatory pain in RA.
2. Two data mining techniques including association rule mining and cluster analysis are performed on the EAHM data collected in this review to discover core herbal material information.

## 2 Methods

The present review will be conducted in accordance with the Preferred Reporting Items for Systematic Reviews and Meta-Analysis 2020 statement.^[14]^ The protocol of this systematic review was prepared according to preferred reporting items for systematic review and meta-analysis protocols (PRISMA-P) 2015,^[15]^ and was pre-registered in PROSPERO (Registration Number: CRD42021273643, available from: https://www.crd.york.ac.uk/prospero/display_record.php?RecordID=273643).

### 2.1 Search strategy

Randomized controlled trials (RCTs) that evaluated the efficacy of EAHM for pain of RA will be searched in the following 11 electronic databases from their inception until October 13, 2021: 4 English databases (PubMed, Cochrane Library, Cumulative Index to Nursing & Allied Health Literature, Excerpta Medica database), 4 Korean databases (Korean Studies Information Service System , Research Information Service System, Oriental Medicine Advanced Searching Integrated System [, Korea Citation Index ), 2 Chinese databases (Chinese National Knowledge Infrastructure Database , Wanfang data), 1 Japanese database citation information by NII. The following Boolean format was used for the search: (rheumatoid arthritis[mesh] OR ((rheumatoid OR reumatoid OR rheumatic OR reumatic OR rheumat∗ OR reumat∗) AND (arthrit∗ OR artrit∗ OR diseas∗ OR condition∗ OR nodule∗))[tw] OR (felty∗ syndrome)[tw] OR (caplan∗ syndrome)[tw] OR (sjogren∗ syndrome)[tw] OR (sicca syndrome)[tw] OR “still∗ disease”[tw] OR “bechterew∗ disease”[tw])) AND ((randomized controlled trial[pt] OR controlled clinical trial[pt] OR random∗[tiab] OR placebo[tiab] OR clinical trials as topic[mesh] OR trial∗[ti]) NOT (animals[mesh] NOT humans[mesh])) AND (“‘Plants, Medicinal”[mesh] OR “Drugs, Chinese Herbal”[mesh] OR “Medicine, Chinese Traditional”[mesh] OR “Medicine, Kampo”[mesh] OR “Medicine, Korean Traditional”[mesh] OR “Herbal Medicine”[mesh] OR “Prescription Drugs”[mesh] OR “traditional Korean medicine”[tiab] OR “traditional Chinese medicine”[tiab] OR “traditional oriental medicine”[tiab] OR “Kampo medicine”[tiab] OR herb∗[tiab] OR decoction∗[tiab] OR botanic∗[tiab]).

### 2.2 Study selection

#### 2.2.1 Type of studies

Only RCTs evaluating the efficacy and safety of oral administration of EAHM for pain of rheumatoid arthritis will be included. There will be no restrictions on language and publication time. Some studies will be excluded if they met the following criteria:

1. not RCT or quasi RCT;
2. the control group was not used or was inappropriate;
3. unrelated to pain of RA;
4. animal experiments;
5. case reports or review; and
6. not published in scientific peer-reviewed journals, including postgraduate theses or dissertations.

**Figure 1:**
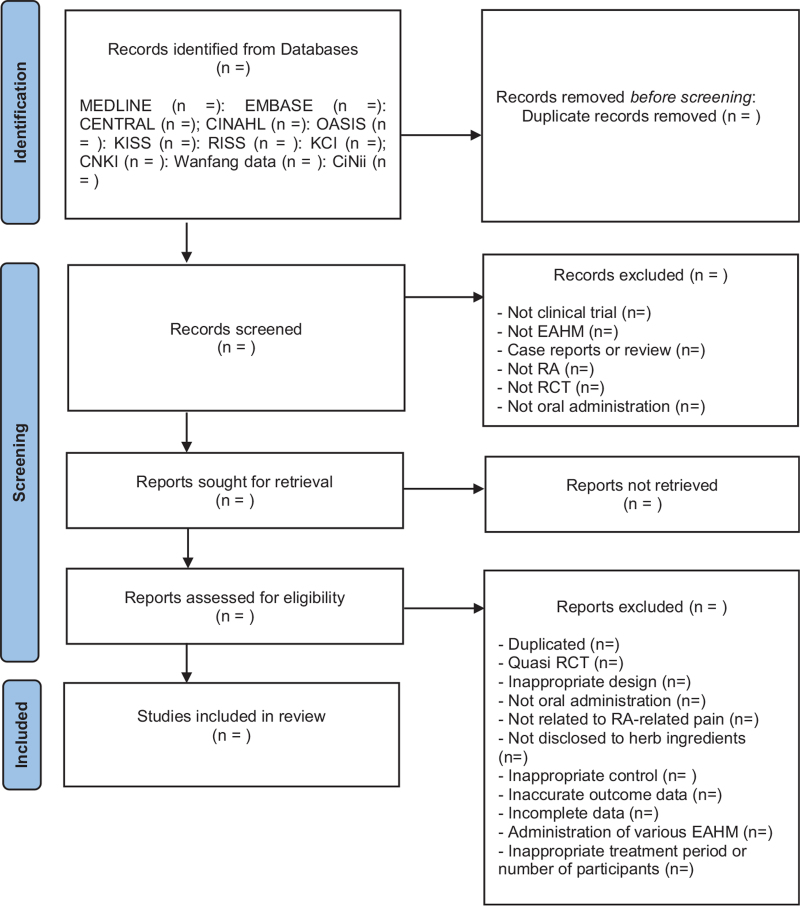
PRISMA 2020 flow diagram.

A PRISMA 2020 flow chart will be produced to show the number of articles identified, screened, included, and excluded (shown in Fig. 1).

#### 2.2.2 Type of participants

Trials will be considered eligible for inclusion if they were conducted in patients with a diagnosis of rheumatoid arthritis, with no restriction on age, gender, or race.

#### 2.2.3 Type of interventions

RCTs that compared EAHM as the active intervention in the treatment group vs CM in the control group will be included. RCTs that used a combination of EAHM and conventional medication as an intervention, on the other hand, will be regarded beyond the scope of the review and will be omitted. All dosage forms of EAHM intervention such as decoction, granule, capsule for the inflammatory pain management in RA will be included. We will exclude studies with a treatment period of less than 4 weeks and less than 30 patients in each group. Studies in which East Asian medical interventions such as acupuncture, massage, or non-drug therapy were only combined in the treatment group will be excluded. On the other hand, even if all other inclusion criteria were satisfied, RCTs for which the exact constituent herbs of the EAHM formulation used as intervention in the study were not identified will be also excluded.

#### 2.2.4 Type of outcome measures

As the primary outcome, continuous pain intensity of RA patients measured with instruments such as the visual analogue scale, numerical pain rating scale (NRS), joint pain index, and joint pain scale of Western Ontario and McMaster Universities Osteoarthritis Index will be adopted. The remission rate of RA pain and inflammation-related symptoms observed according to explicit criteria will be also selected as the primary outcome. Meanwhile, the tender joint count measured by individual RCTs will be employed as the primary endpoint.

The first group of the secondary outcome will be to evaluate the inflammatory status accompanying pain in RA patients. Accordingly, outcomes swollen joint count, erythrocyte sedimentation rate, and C-reactive protein will be selected as a series of index groups to evaluate inflammatory findings. On the other hand, the second group of secondary outcomes is an index that can evaluate the overall related symptoms that may accompany inflammation and pain of RA and the patient's physical condition, disease activity score 28 (DAS28), health assessment questionnaire, a 20% improvement in response rate as defined by the American College of Rheumatology will be selected. Finally, whether EAHM monotherapy secured safety compared to CM will be also set as an important evaluation goal, and for this purpose, the incidence rate of adverse events reported by each RCT will be set as one of the secondary outcomes. In the case of this indicator, statistical comparison will be performed according to the type of individual CM used as a comparator.

#### 2.2.5 Data extraction

Titles and abstracts of potentially eligible studies will be independently screened by 2 investigators according to the search strategy mentioned above. For the selected studies, 2 reviewers will independently collect the following information from the included studies title, author's name, clinical trial conducted country, diagnostic criteria, trial design publication year, sample size, participant age, sex distribution, interventions in the treatment and comparators, treatment duration, outcome index, reported adverse event, and composition with dosage of EAHM. Any discrepancies will be resolved through discussion among researchers.

#### 2.2.6 Methodological quality assessment

The methodological quality of each included study will be independently evaluated by two investigators according to Rob 2.0, a modified tool for risk of bias in randomized trials.^[16]^ Rob 2.0 consists of 5 domains: bias arising from the randomization process, bias deviating from the intended intervention, bias due to omission of outcome data, and bias in the selection of reported outcomes. Methodological quality is “high risk of bias”, “ It will be assessed on 3 levels of “low risk of bias” and “some concerns”, and discrepancies between raters will be resolved through discussion.

#### 2.2.7 Statistical analysis

##### 2.2.7.1 Evidence synthesis

Evidence synthesis of included studies with available data will be performed by calculating the effect size and 95% CI using only the random effect model. Heterogeneity will be considered statistically significant when the *P* value based on the χ^2^ test is less than 0.10 or *I*^2^ is is greater than or equal to 50%. Two-sided *P*<.05 will be considered statistically significant. Statistical synthesis of individual research results will be performed in the Software R (Version 4.1.1, R Foundation for Statistical Computing, Vienna, Austria) and R studio program (Version 1.4.1106, Integrated Development for R. R Studio, PBC, Boston, MA) using the default settings of the “meta” package and ‘metafor’ package.^[17]^ In this review, in order to effectively reveal the exact value of the effect size without relying only on the *P* < .05 significance threshold in the interpretation of the primary outcome synthesis result, a drapery plot will be additionally illustrated along with the forest plot.^[18]^ The studies will be grouped according to type of intervention such as EAHM and comparator such as CM. Relative risk (RR) and 95% confidence interval (CI) were calculated for remission rate. For continuous pain intensity, standardized mean difference, and 95% CIs will be calculated to integrate the results of several types of indicators for the same measurement target. Mean difference and 95% CIs will be calculated for tender joint count, swollen joint count, erythrocyte sedimentation rate, and C-reactive protein. Adverse events will be calculated using the odds ratio. If heterogeneity was observed in the synthesized meta-analysis results for the primary outcome, subgroup analysis will be performed by determining the scope of additional analysis for the following 5 items in advance to identify the cause:

1. type of comparator,
2. diagnostic criteria,
3. treatment duration,
4. source of investigational medication,
5. type of preparation.

In order to distinguish publication bias, a contour-enhanced funnel plot will be used for the outcome that included the most studies.^[19]^ For the asymmetry on the visually confirmed funnel plot, Egger test^[20]^ and Begg test^[21]^ will be additionally performed to specifically confirm the existence of publication bias.

##### 2.2.7.2 Association rule mining

By analyzing the constituent herb data of EAHM collected from the included study, the potential association rules of core herb combinations will be explored. Before proceeding with this analysis, preliminary information for data mining will be extracted by first analyzing the frequency of individual herbs. The R studio program (Version 1.4.1106, Integrated Development for R. R Studio, PBC, Boston, MA) will be used for the Apriori association rule analysis and plot production. A data fit will be done by using “arules” package in R studio.^[22]^ The function of the R package “arulesViz” will be applied to generate graphical presentations according to the results.^[23]^ Mining of frequent herb item sets and association rules will be performed according to the Apriori algorithm method for discovering meaningful relationships between variables in a large database.^[24]^

In the Apriori algorithm, support, confidence, and lift are the main metrics for measuring association. A rule is defined as an expression X⇒Y where X, Y ⊆ I and X∩Y=ϕ. The herb X and herb Y are called antecedent (left hand side, LHS) and consequent (right hand side, RHS) of the rules. Association rules are rules which surpass researcher-specified minimum support and minimum confidence thresholds. The support, supp(X), of an itemset X is a measure of importance defined as the proportion of transactions in the dataset which contain the itemset. The confidence of a rule is defined as conf(X⇒Y) = supp(X∪Y)/supp(X), measuring how likely it is to see herb Y in a transaction containing herb X. An association rule X⇒Y needs to satisfy supp(X∪Y)≥σ and conf(X⇒Y)≥δ, where σ and δ are the minimum support and minimum confidence, respectively. Confidence can be interpreted as an estimate of the probability P(Y|X), the probability of finding the RHS of the rule in transactions under the condition these transactions also contain the LHS. Lift of a rule is defined as lift(X⇒Y) = (supp(X∪Y)/supp(X)). Support is a measure to evaluate the usefulness of the association rule and is the proportion of prescriptions containing a specific herb combination pattern in the total EAHM prescription. When the confidence is close to 1, herb A and herb B are irrelevant because they are close to independence in probability. Meanwhile, if the lift value is large, the correlation is interpreted as strong. In this review, the association rules will be identified based on the minimum values for support and confidence being 20% and 80%, respectively. And it will be deriving the core herb pattern and its constituent herbs that show the most obvious association among them.

##### 2.2.7.3 Hierarchical agglomerative clustering

Hierarchical cluster analysis will be used to understand the structure of the EAHM formulation used in individual studies. The analysis will be utilized in this study is agglomerative clustering, in which each observation is initially considered as a cluster of its own (leaf). Then, the most similar clusters are successfully merged until there is just one single big cluster (root). Dissimilarity between individual herb constituents will be considered as an individual distance, and the Euclidian distance will be used as a measure of this. This corresponds to the shortest distance when it is assumed that the difference between each characteristic value is expressed on the coordinate plane.

#### 2.2.8 Quality of evidence according to outcome measurements

The overall quality of evidence for each outcome will be evaluated according to the Grading of Recommendations Assessment, Development, and Evaluation pro.^[25]^ The Grading Quality of Evidence and Strength of Recommendations in Clinical Practice Guidelines assessment evaluates the overall quality of evidence in 4 levels: very low, low, moderate, and high. The level of evidence is lowered according to factors such as risk of bias, inconsistency, indirectness, imprecision, and publication bias, respectively.

## 3 Amendments

If there is a significant modification or change of this protocol, the details and date of all amendments will be described in the final report.

## 4 Ethics and dissemination

In the process of implementing this systematic review, personal information will not be disclosed or published. This review will not infringe the rights of the subjects. Since it is not a clinical study that directly recruited subjects, ethical approval is not possible. The results of this study will be reported in a peer-reviewed scientific journal.

## 5 Discussion

This systematic review will provide comprehensive information on the efficacy and safety of EAHM for inflammatory pain of RA. Several prior meta-analyses on the effects of East Asian herbal therapy on RA have previously been published.^[26–31]^ However, existing studies only dealt with the efficacy of a single herb or herbal formulation, or even if the overall EAHM was investigated, the number of included studies was insufficient, so it was difficult to see that a sufficiently extensive investigation was conducted to be used for drug discovery. On the other hand, even if meta-analysis of the overall EAHM is performed, it is difficult to derive useful drug information simply by examining the pooling effect. The goal of using EAHM is to optimize the synergistic impact that happens when it's given as a mixed prescription.^[32,33]^ As a result, even when a systematic literature analysis of numerous EAHM clinical trials for the same condition is conducted, the prescription formulation and dose of the included studies are frequently different. In fact, seeing numerous meta-analyses of herbal medicine as a treatment as a statistical synthesis of a single intervention is problematic. For these reasons, it is difficult to identify which of the many herb-related information reflected in the review is useful information.

In such a situation, the evidence according to this review will provide more comprehensive and specific information to both clinicians who manage pain of RA. In addition, it is expected that the derivation of core herb material information using data mining techniques can be used as a hypothesis worthy of follow-up research in the development of new drugs related to the subject.

## Author contributions

**Conceptualization:** Hee-Geun Jo, Ho-Sueb Song, Donghun Lee.

**Data curation:** Hee-Geun Jo.

**Formal analysis:** Hee-Geun Jo.

**Funding acquisition:** Ho-Sueb Song, Donghun Lee.
